# What over 1,000,000 participants tell us about online research protocols

**DOI:** 10.3389/fnhum.2023.1228365

**Published:** 2023-07-06

**Authors:** Johanna Tomczak, Andrew Gordon, Jamie Adams, Jade S. Pickering, Nick Hodges, Jo K. Evershed

**Affiliations:** ^1^Gorilla Experiment Builder, Cauldron Science, St Johns Innovation Centre, Cambridge, United Kingdom; ^2^Prolific, London, United Kingdom

**Keywords:** online research, online methods, survey research methods, behavioral research methods, experimental psychology, crowdsourcing, research methodology

## Abstract

With the ever-increasing adoption of tools for online research, for the first time we have visibility on macro-level trends in research that were previously unattainable. However, until now this data has been siloed within company databases and unavailable to researchers. Between them, the online study creation and hosting tool Gorilla Experiment Builder and the recruitment platform Prolific hold metadata gleaned from millions of participants and over half a million studies. We analyzed a subset of this data (over 1 million participants and half a million studies) to reveal critical information about the current state of the online research landscape that researchers can use to inform their own study planning and execution. We analyzed this data to discover basic benchmarking statistics about online research that all researchers conducting their work online may be interested to know. In doing so, we identified insights related to: the typical study length, average completion rates within studies, the most frequent sample sizes, the most popular participant filters, and gross participant activity levels. We present this data in the hope that it can be used to inform research choices going forward and provide a snapshot of the current state of online research.

## 1. Introduction

In recent years, the use of online platforms for participant recruitment, experiment building, and testing has become increasingly popular in scientific behavioral research (Sauter et al., 2020). The ease and convenience of recruiting participants through the internet have made it a common method for collecting large and diverse data sets quickly (Chetverikov and Upravitelev, 2016), or for facilitating experimental designs that may be too complex, too impractical, or too costly for traditional in-person testing. By Zhou and Fishbach (2016), the number of studies published in major journals using only online recruitment platforms (such as Amazon MTurk) had begun to eclipse those using traditional methods (Goodman and Paolacci, 2017), and since then it has been reported that the number of online studies has increased at least tenfold (Walter et al., 2019; Lu et al., 2022).

This rapid acceleration in the use of online methods for research has been driven by a number of likely factors such as increasing access to technology, a greater awareness of the opportunities that remote testing provides, and the proliferation of tools designed to facilitate online research. Indeed, online research tools now exist for traditional reaction time studies, user experience studies, user interface studies, AI model training, multiplayer studies, consumer behavior studies, and gamified studies, going far beyond traditional surveys. Additionally, global events such as the COVID-19 pandemic at the beginning of 2020 have also played a significant role in this increased adoption by forcing the majority of researchers to abandon lab-based recruitment entirely (Rashid and Yadav, 2020; De Man et al., 2021). Now, although the COVID-19 pandemic has abated and labs are once again open for in-person testing in most countries, researchers have continued to utilize online methods for research, highlighting the recognition and embedding of online participant recruitment and testing as a convenient, cost-effective, and efficient solution for researchers across a variety of disciplines.

The exponential growth of online research methods also affords a unique opportunity not previously available to researchers - information about macro-level trends and standards in online research. With the advent of platforms for online research, we now have the ability to access metadata from thousands of studies, across dozens of disciplines, and generate insights related to trends in scientific research that will likely be invaluable to researchers when constructing grant applications and funding requests. However, despite the wealth of data now being accessible, it remains hidden within company data warehouses and thus little to no information has been made publicly available to researchers. Science is therefore at the real risk of moving from the file-drawer problem (Rosenthal, 1979) to the data-warehouse problem.

We aim to address this empirical lacuna by presenting and analyzing metadata from our own platforms associated with online research: Prolific (Peer et al., 2017; a participant recruitment platform for online research; Palan and Schitter, 2018)^1^ and Gorilla Experiment Builder (a platform for designing and hosting studies with surveys, reaction-time tasks, games, multiplayer tasks and more; Anwyl-Irvine et al., 2020, 2021).^2^ Although used for different steps in the research process, recruitment and experiment design, respectively, these two platforms hold metadata gleaned from well over 1 million participants and from over half a million studies conducted online. Our focus is providing insights into key study metrics that will benefit researchers conducting online research, as well as highlighting the potential for continued research in this area. As researchers submit grant and ethics applications with *a priori* numbers of study parameters, we would like to facilitate the process by providing a summary of key metrics in online research that could help inform decisions researchers make when designing their study. In the research cycle, researchers usually start by designing our study, before they move on to the recruitment of participants and finally data collection. Hence, we decided to follow the typical research process in the way we report our metrics, choosing to focus on sample size and study length, completion rates, participant filters, and aggregated participant activity levels.

## 2. Method

### 2.1. Measure selection

#### 2.1.1. Sample size and study length

Sample size and study length are two critical factors that determine not only the validity of the research (it is well-attested that small sample sizes reduce statistical validity of a study; Faber and Fonseca, 2014), but also considerations and justifications when applying for funding. Online research methods address the former issue by providing researchers with access to potential larger samples and making it easier to run longer studies than traditional in-person research. Knowing the distribution of study lengths helps researchers to take into account the typical length of studies participants are willing to take part in, and the consideration of attrition with longer studies. In online research, this becomes particularly important because participants are typically paid pro-rata for their time, and therefore overestimation of study length can quickly lead to a higher financial burden for the researcher. In addition, knowing the range of sample sizes for studies conducted online can provide researchers with critical information about how their sample size relates to current boundaries of successful data collection.

#### 2.1.2. Participant filters

With the latest generation of online research platforms, researchers now have the ability to filter their participants beforehand to ensure that their sample conforms to certain desired criteria (see Peer et al., 2021). Prolific allows you to filter participants on over 250 participant characteristics (e.g., location, age, ethnicity) which allows researchers to select those that are important for their work. It is not uncommon to seek homogeneity on some characteristics and heterogeneity on other characteristics, and online recruitment allows for the opportunity to diversify samples of participants. However, although some filtering recommendations exist–for example, Peer et al. (2021) recommend filtering for participants with a high approval rate and high number of previous submissions as key for increasing data quality–filters are mostly used without respect to accepted standards and norms. Approval rate refers to the ratio of approved submissions to rejected submissions and is used by researchers as a measure of how a participant has performed in research studies previously. Understanding the most commonly used filters allows researchers to make sensible decisions about how to get high quality data while recruiting sufficiently diverse participants.

#### 2.1.3. Completion rates

Average completion rate (analogous to the concept of “retention rate”) across multiple studies can provide valuable data to researchers when it comes to planning their sampling and expected study cost (Göritz, 2014). Understanding the typical completion rate helps researchers better design studies and set recruitment targets to account for anticipated dropout. Incomplete data could reduce statistical power if not accounted for and can bias results, as dropout rates can mean smaller sample sizes, which makes it harder to detect true effects, and these also affect statistical validity, leading to unequal attrition from different conditions. Additionally, funders and ethics boards may consider within-study attrition when evaluating research proposals and practices. Accurate completion rates coupled with sample size and study length help researchers budget appropriately so that they get the data quality they need to deliver the requirements of their research project.

#### 2.1.4. Participant activity cycles

With asynchronous online recruitment methods, researchers are given access to larger potential participant pools than ever before and participants are now able to complete multiple surveys consecutively. This has led to a dramatic increase in the speed at which participation occurs and therefore the rate at which a research study can be completed, making it more important to know when a study is most likely to be able to access its intended sample in order to better plan research. Giving researchers a better understanding of when the participant marketplace has the most liquidity (the size of the available participant pool) allows them to distribute their studies during those times. This may not only help increase the response rate of the study, but also make the data more representative as it is less likely to be biased toward those who happen to be active during a particular time.

### 2.2. Data collection and exclusions

#### 2.2.1. Prolific data

We collected data on every study published on Prolific in 2022. In total, 185,302 studies were published in 2022. We excluded studies from further analysis based on researchers’ estimated time for completion (i.e., the anticipated study completion time set by researchers on Prolific). Specifically, studies with an estimated completion time under 2 min (*n* = 14,132) were excluded as they were very likely to be custom screening studies (i.e., studies where a researcher is simply checking the availability of a certain sample) and thus likely did not represent actual research being carried out.

We also excluded any studies that only recruited a single participant (*n* = 19,367), as these were highly likely to represent researchers testing their own studies as a participant and are therefore not representative of actual research. It is worth noting that there was some degree of overlap between very short studies and those with only a single participant. No further exclusions were carried out, leaving us with 152,127 studies for our final analysis.

To analyze pre-screener usage we removed three potential screeners from consideration: Participant Allow List, Participant Block List, and Previous Study Block List. These screeners are available to all researchers, but will be used in different ways for each (for example, a researcher may use a “Participant Allow List” for a longitudinal study or to re-invite participants to a study) - thus they are not homogenous across all studies and we opted not to include them.

#### 2.2.2. Gorilla data

We selected the most recent 1 million participants who took part in any research study on Gorilla before the end of the year 2022. In total, these participants comprised data from 14,940 studies. When looking at study length, we examined data only from participants who reached the end of an experiment. Next, outliers were removed by excluding participants with a completion time of less than 2 min (in our experience, these are likely incomplete experiments used for piloting purposes), leaving a total of 639,937 participants for analyses. Similarly, outliers were also removed when examining sample size by excluding studies with <5 participants; 11,656 studies remained for the analysis.

## 3. Results

### 3.1. Sample size and study length

Across the 152,127 studies published on Prolific that remained after exclusions had been applied, the median sample size was 50 participants (Q1 = 12, Q3 = 180). We note however, that the tails on the distribution were large and that 9,345 studies with *N* ≥ 500 were conducted in 2022, demonstrating that large samples are being collected routinely on the platform (we include an analysis of these large studies in the Supplementary material). A similar skew was seen in Gorilla data, of the 1 million participants tested on Gorilla, 61% were in small studies (*N* < 250), 15% were in medium studies (251–500 N) and 24% were part of large studies (*N* > 500), demonstrating that large samples are also evident on this platform despite the low averages.

For Prolific data, we also analyzed study size split by survey versus experimentally focused platforms to examine whether study size was related to the platform focus. We defined survey platforms as any platform that is predominantly used for survey/questionnaire research (this group included: Qualtrics, SurveyMonkey, Typeform, SurveyGizmo, SoSciSurvey, Alchemer, Cognito Forms, Google Forms, Measuring U, SmartSurvey, and Unipark), and experiment platforms as any platform used primarily for behavioral science and psychological experimentation (this group included: Gorilla, Cognition, Millisecond/Inquisit, Pavlovia, and PsyToolKit).

Experiment-focused platforms had a lower median sample size (*N* = 22) in comparison to survey-focused platforms (*N* = 50). This was backed up by a separate analysis of sample size on Gorilla (a platform that primarily focuses on experiments rather than surveys); across the 11,656 studies remaining after outlier removal, a similar median sample size (*N* = 40, Q1 = 16, Q3 = 89) was evident.

**FIGURE 1:**
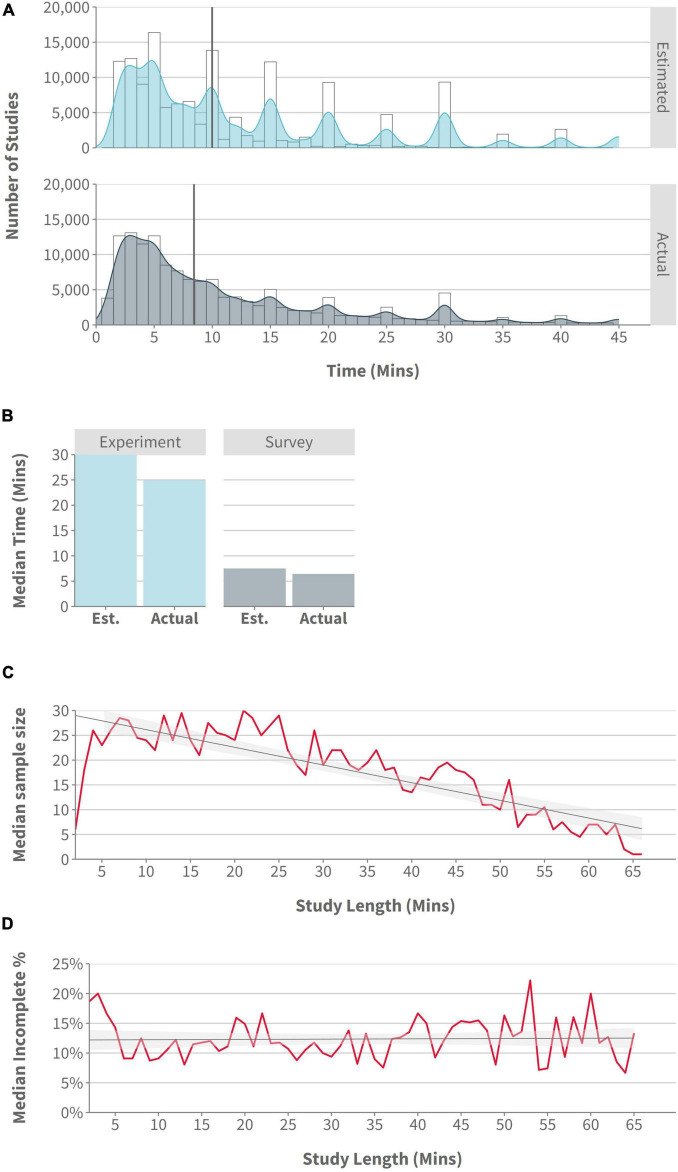
**(A)** Histogram/density: number of studies by estimated and actual study length, with a cutoff of 45 min to improve data visualization (Prolific data); **(B)** Bar plot: median estimated and actual study length by Survey and Experiment platforms (Prolific data); **(C)** Line plot: median sample size by study length, with a cutoff of 65 min to improve data visualization (rounded to nearest minute; Gorilla data); **(D)** Line plot:% of incomplete participants by study length, with a cutoff of 65 min to improve data visualization (rounded to nearest minute; Gorilla data).

Regarding study length, the median study length estimated by researchers on Prolific was 10 min (Q1 = 5, Q3 = 20), while the median actual study length was 8.43 min (Q1 = 4.19, Q3 = 18.14). These data indicated that, on average, researchers are over-estimating how long their studies take participants to complete (see Figure 1A; for the purposes of visualization, we removed any study with estimated or actual completion times >50 min). We also note that 6,789 studies had an estimated completion time ≥60 min (we include an analysis of these long studies in the Supplementary material). For Gorilla data, a median study length of 17.65 min (Q1 = 9 min, Q3 = 32.5 min) was evident.

Experiment-focused platforms had a higher estimated and actual completion time (30 and 24.85 min, respectively) than did survey platforms (7.5 and 6.42 min, respectively) (see Figure 1B). For studies specifically using Gorilla and Prolific, the median estimated study length was 25 min, and the median actual study length was 21.92 min. Regardless of the type of platform used, there remained a tendency for researchers to overestimate completion times–though the difference between estimated and actual completion times was noticeably larger on experiment-focused platforms (5.15 min), than on survey platforms (1.08 min).

Using Gorilla data, we also looked at the relationship between study length and sample size on Gorilla and found median sample sizes decreased as study length increased (see Figure 1C). Despite this, longer studies did not appear to increase the proportion of incomplete participants (see Figure 1D).

The highest median sample size was found for recruitment platforms (e.g., Prolific or MTurk) (*N* = 40.5), followed by studies that distribute a simple link on social media or by email for completely anonymous recruitment (*N* = 26), then ID-based recruitment methods (*N* = 20; ID based methods are controlled recruitment options where researchers want to keep track of which participants have completed the study so they can join the data to a secondary data set, keep track of students for student credit, or because they want the participant to be able to log back in again for a longitudinal protocol), and finally the pilot studies (*N* = 8). In terms of study length, ID-based methods were generally used for the longest studies (38.0 min), followed by recruitment platforms (23.2 min, closely mapping findings from Prolific data), and finally studies advertised via email or social media with a simple URL (18.2 min).

### 3.2. Participant filters

The Prolific data shows that country of residence was the most popular screener used by researchers regardless of territory, appearing in almost three-times as many studies as the next most popular option (see Table 1). This is likely due to the fact that on Prolific researchers are motivated to select a specific country for their sample to avoid the study being released to all available countries simultaneously and creating potential confounds from the resulting demographics. Age was the second most popular screener and interestingly was almost twice as likely to be selected in the UK when compared to all other territories. Quality-related screeners such as approval rate, first language, fluent languages, and number of previous submissions also featured heavily in the top ten. We discuss the implications of this below.

**TABLE 1:** The proportion of studies using the ten most popular screeners on Prolific.

Screener	Overall	UK	US	EU	ROW
Current country of residence	73.77%	72.22%	81.08%	62.19%	74.58%
Age	25.22%	41.04%	20.41%	23.87%	24.68%
Approval rate	24.63%	20.01%	25.82%	24%	26.84%
First language	23.11%	22.07%	16.11%	31.55%	28.66%
Fluent languages	21.22%	28.88%	20.07%	21.79%	17.46%
Nationality	16.34%	13.2%	16.07%	15.41%	20.51%
Number of previous submissions	11.03%	8.3%	12.03%	11.68%	10.59%
Sex	6.09%	8.7%	4.47%*	5.48%	8.06%
Employment status	5.30%	6.31%	5.83%	3.59%*	5.48%*
Highest education level completed	4.7%	8.13%	3.6%*	5.32%	3.33%*

The “Overall” column represents the proportion of use across all studies in 2022, while the remaining columns represent the proportion of use for that screener in different territories. ROW, rest of the world.

*Screener was not in the top 10 for that territory.

### 3.3. Completion rates

**FIGURE 2:**
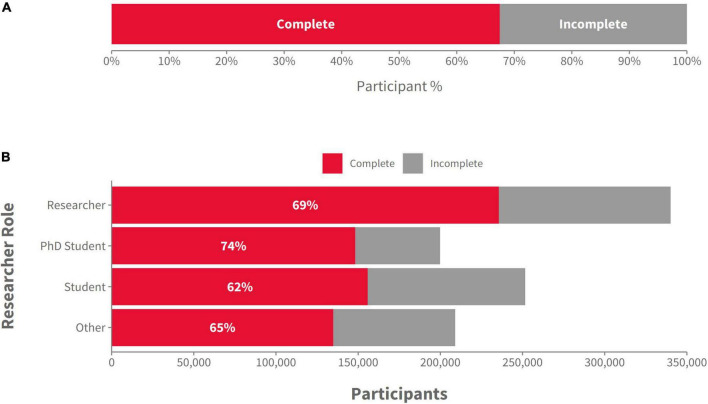
**(A)** The proportion of “complete” and “incomplete” participants in the sample of 1,000,000 participants, across all recruitment methods and researcher types (Gorilla data); **(B)** The number (and proportion) of “complete” and “incomplete” participants by the role of the researcher (Gorilla data).

To analyze average completion rates, we exclusively used the more granular study-level Gorilla metadata and compared the ratio of participants that completed a study with the total number of participants recruited. On average, 67.5% of participants recruited went on to complete a study successfully, meaning that the remaining 32.5% either dropped out during the experiment or were excluded by the researcher during their participation for example for not meeting certain criteria in a screening survey or for failing attention checks (Figure 2A). We were also interested in whether there were differences in average completion rates by recruitment method. For the purposes of this analysis, we removed participants who had not yet completed an experiment or been rejected by the researcher, as they might be taking part in a longitudinal study and cannot be considered as either complete or incomplete. The data revealed that studies with ID-based recruitment methods had the highest completion rates (84%), followed closely by pilot studies (78%), studies done through recruitment platforms (76%), and finally recruitment through a simple link/URL (56%) which is often used for crowdsourcing on social media. In addition, we wanted to see how the researcher’s role affected the quantity and completion rate of participants (see Figure 2B). Students (which includes undergraduate and Master students) had the lowest completion rates, probably linked to their recruitment methods, while Ph.D students had the highest completion rates, although recruiting less participants in total than researchers.

### 3.4. Participant activity cycles

**FIGURE 3:**
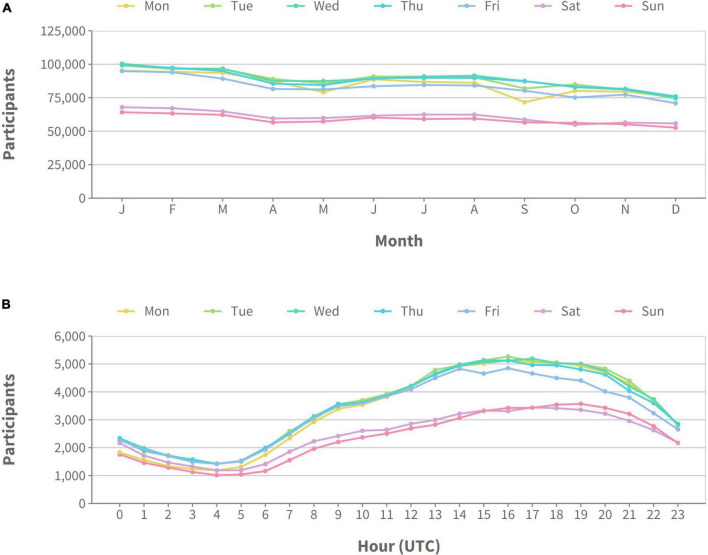
Number of active participants on the Prolific platform during 2022. Different lines represent different days of the week. Plot **(A)** shows activity averaged across months of the year, while plot **(B)** shows activity averaged across hours of the day (all times shown in UTC).

We examined participant activity and availability in Prolific data throughout the year in two separate ways. We first looked at gross activity, that is, the total number of participants online for any given day averaged across months of the year. Figure 3A shows these gross activity levels overall and split by territory. The overall trend shows that, on average, there are ∼30,000–40,000 fewer participants active at the weekend compared to mid-week). Month-to-month variation was more random, however, there was a general trend toward higher activity at the beginning of the year, a slight increase during the summer months, and then less activity at the end of the year. This general pattern held true for all territories, however, overall activity was reduced in the EU and the rest of the world (ROW) compared to the UK (which had the highest) and the US. Participants are most active on Prolific in the afternoon (UTC time) regardless of the day of the week, but were more active overall on weekdays (see Figure 3B). This pattern didn’t change when we split the data by territory.

## 4. Discussion

As suggested by Birnbaum (2021), technical and methodological considerations around online research are not usually addressed in textbooks given the rapid rate of technological advancements. The data we present here provides several important insights into the current state of online research. A number of trends emerge from the data that suggest researchers are, by and large, still recruiting relatively small samples and making adequate (but not complete) use of demographic filters. Looking at macro-level trends, we also identified a number of predictors of participant completion and gained insight into marketplace liquidity on recruitment platforms. These findings are discussed in more detail below.

We found that both study length and sample size appear to vary with the type of recruitment method used: shorter studies are more likely to be crowdsourced, while longer studies are more likely to use professional recruitment platforms such as Prolific. On the whole, studies using survey-based platforms (such as Qualtrics) also had a considerably higher number of participants and a longer estimated time than studies using experiment-focused platforms (such as Gorilla). It is noteworthy that the median sample size for survey-based platforms was close to the minimum sample size typically recommended for survey-based research (Barlett et al., 2001), suggesting that the ability to recruit more participants online has not necessarily translated to adequate and justifiable sample sizes for survey-based research (see Lakens, 2022 for more information on justifying sample sizes). We caveat this interpretation however, by highlighting that ∼5.5% of studies on Prolific and ∼24% of participants on Gorilla were studies with a sample size over 500, which clearly shows that despite the median being on the low side, researchers can and are recruiting large online samples. As low power due to inadequately small sample sizes contributes to research waste (Button et al., 2013), the potential for researchers to collect high quality data from large sample sizes quickly with online research platforms is particularly encouraging.

We were also interested in average completion rates, to provide researchers with a baseline they can use to plan their sampling more effectively. In Gorilla, there was no evidence of increased attrition in longer studies. We found the highest completion rates for ID-based recruitment methods (84%) which, as stated above, give researchers more control during recruitment and data collection. We suggest that this could also be due to a social effect, as participants might be more known to the researchers and not anonymous. The lowest overall completion rates were found for simple link recruitment and we tentatively attribute this to the fact that recruitment through social media usually only includes low incentives for participants. However, as dropout rates vary across populations, topics, and study designs, researchers still need to evaluate expected attrition for their particular work. Studies that specifically examine attrition can help point to the factors that most influence dropout, so researchers know where to focus their efforts to reduce it (e.g., McCambridge et al., 2011). Interestingly, we found that although there are more participants recruited by early career or senior researchers, students still manage to collect many high quality data and are able to power their studies appropriately.

We also looked at the data with a focus on participant selection we found that country of residence and age were the two most popular filters used by researchers across all studies. Interestingly however, quality-related screeners such as approval rate, first language, fluent languages, and number of previous submissions are highly popular options, and may reflect the growing need to recruit only the best participants within any given pool (Peer et al., 2021; Douglas et al., 2023) while addressing the need to balance the sample between experienced and naive participants (Rodd, 2023). While we accept the criteria for participant filters will depend on the research question and the goals of the study, we hope that these data serve to spotlight the importance researchers place upon quality-screening in online samples, ensuring convenience does not come at the cost of quality or integrity.

Regarding when a researcher should sample, we found participants were considerably more active during the week than at weekends, and there was a steady decline in participant activity levels throughout the year (we refrain from over-interpreting this decline as this may have been unique to 2022). At the daily level, participant activity was closely correlated with typical working hours with a tendency for higher activity in the afternoon. When planning recruitment, we encourage researchers to think about the activity levels we present here, their studies are distributed at a time they think is most suitable to reach the largest number of participants in their particular location.

The current metadata has some limitations, as studies designed on Gorilla could also theoretically be used in the lab, and, for both platforms, we do not have easy access to whether a study was designed as a pilot or as the final experiment. Separating real and pilot data may show much larger samples for real studies, if every real study currently also has a small pilot study. Adding this information to study analytics in the future could help researchers better understand trends in online research and provide more thorough interpretations of the effects we observed. Additionally, whilst we can report on how researchers are currently running their studies, this should not be interpreted as a recommendation, as sample sizes and study length should be calculated on a per study basis by taking into account power analyses, availability of the sample, and research budget. It does, however, demonstrate the scope of what is possible.

Our data shows that large sample sizes are possible, and that completion rates are promising. When recruiting participants through ID-based methods, for example when the participant is known to the researcher or has an account on a platform such as Prolific (especially when bolstered by quality filters), completion rates are high (76–84%) and seem to depend more on the recruitment method than other factors such as the length of the study session or whether the study is longitudinal/multi-part. Whilst the data presented here is useful for planning studies and writing grant applications we, anecdotally, also observe a high number of undergraduate students successfully running experiments independently with Gorilla each year, and so we believe that powerful tools for online research can be embedded in teaching and research projects.

## Data availability statement

The datasets presented in this study can be found in online repositories. The names of the repository/repositories and accession number(s) can be found below: https://osf.io/hk59y/.

## Author contributions

AG, NH, and JE contributed to the conception and design of the study. AG and JA analyzed the data. AG, JA, JP, and JT interpreted the data. JA created the data visualizations. AG and JT wrote the first draft of the manuscript. All authors revised the manuscript and approved the submitted version.

## Footnotes

1
www.prolific.co

2
www.gorilla.sc
