# Supplementary material for: What over 1,000,000 participants tell us about online research protocols
Source: Front Hum Neurosci. 2023 Jul 6;17:1228365. doi: 10.3389/fnhum.2023.1228365 (PMC10357382; doi:10.3389/fnhum.2023.1228365)
Supplement: Supplementary file 1 [file Table_1.DOCX]

Supplementary Material

What over 1,000,000 participants tells us about online research protocols

**Johanna Tomczak^1^†, Andrew Gordon^2^†, Jamie Adams^1^, Jade S. Pickering^1^, Nick Hodges^1^, Jo K. Evershed^1^***

†These authors contributed equally to this work and share first authorship

^1^Gorilla Experiment Builder, Cauldron Science, St Johns Innovation Centre, Cambridge, UK

^2^Prolific, London, UK

*** Correspondence:**Jo K. Evershed
[jo@gorilla.sc](mailto:jo@gorilla.sc)

# Supplementary Data

In addition to our gross analysis of study length and sample size we also ran a separate analysis to discover more about the very long or very large studies that are being run on Prolific. This data may be useful to any researchers seeking to conduct these types of studies online.

## Large study analysis

Below we provide summary data for studies with an N ≥ 500. In total, 9,345 studies met this criterion.

For this subset of studies, the median estimated completion time was 5 minutes (IQR = 7), while the median actual completion time was 4.5 minutes (IQR = 5.93). This reflected a similar pattern to what we observed in the main analysis, however the difference between estimated and actual was notably shorter. The median sample size was 700 (IQR = 429).

We also analyzed the top participant filters that researchers used to facilitate studies with a large sample size (see table S1). The pattern of top filters was remarkably similar to those used in our overall study analysis, however for these larger studies the ‘responses approved’ filter was considerably more common. This possibly indicates that for these larger studies researchers are preferentially recruiting participants with a stronger track record of approvals on the platform.

## Long study analysis

Below we provide summary data for studies with an estimated completion time ≥ 60 minutes. In total, 6,789 studies met this criterion.

For this subset of studies, the median estimated completion time was 60 minutes (IQR = 15), and the median actual completion time was identical at 60 minutes (IQR = 18.33). This subset of studies therefore did not display the same bias towards overestimation as other studies being conducted on the platform. The median sample size was 12 (IQR = 28), notably lower than the median sample size observed in the overall analysis, suggesting that longer studies typically recruited fewer participants.

We also analyzed the top participant filters that researchers used to facilitate studies with a longer duration (see table S2). The pattern of top filters was similar to those observed in both the overall study analysis and the large study analysis, however interestingly both ‘Vision’ and ‘Mild cognitive impairment/Dementia’ featured in 13.62% and 7.16% of long studies respectively. We refrain from an over-interpretation of these findings; however they may point to longer studies often being used for clinically-orientated work.

# Supplementary Figures and Tables

**Table S1:** The ten most popular filters used for studies with a sample size of N ≥ 500. Percentages represent the number of large studies using each filter.

| **Filter** | **Proportion of Studies** |
| --- | --- |
| **Current Country of Residence** | 85.79% |
| **Approval Rate** | 31.05% |
| **Nationality** | 21.1% |
| **Fluent Languages** | 20.15% |
| **First Language** | 16.27% |
| **Age** | 15.62% |
| **Responses Approved** | 14.14% |
| **Employment Status** | 10.83% |
| **Highest Education Level Completed** | 2.89% |
| **Ethnicity** | 2.58% |

**Table S2:** The ten most popular filters used for studies with an estimated completion time ≥ 60 minutes. Percentages represent the number of large studies using each filter.

| **Filter** | **Proportion of Studies** |
| --- | --- |
| **Current Country of Residence** | 65.21% |
| **Age** | 47.9% |
| **Fluent Languages** | 34.69% |
| **First Language** | 32.08% |
| **Approval Rate** | 28.77% |
| **Responses Approved** | 14.45% |
| **Vision** | 13.37% |
| **Nationality** | 11.74% |
| **Highest Education Level Completed** | 7.47% |
| **Mild cognitive impairment/Dementia** | 7.01% |
